# Effect of Relative Humidity on Quality and Metabolite Profiles of *Perilla frutescens* Seed Powder During Storage

**DOI:** 10.3390/molecules30183662

**Published:** 2025-09-09

**Authors:** Dong-Shin Kim, Kyo-Yeon Lee, Ji Yeong Park, Yejin Son, Suyeon Gu, Sung-Gil Choi, Myoung-Hee Lee, Hyun-Jin Kim

**Affiliations:** 1 National Institute of Horticultural and Herbal Science, Rural Development Administration, Wanju 55365, Republic of Korea; dskim3309@korea.kr; 2 Division of Applied Life Sciences (BK21 Four), Gyeongsang National University, 501 Jinjudaero, Jinju 52828, Republic of Korea; leeyeon0511@gnu.ac.kr (K.-Y.L.); yejin7739@naver.com (Y.S.); sgchoi@gnu.ac.kr (S.-G.C.); 3 National Institute of Agricultural Science, Rural Development Administration, Wanju 55365, Republic of Korea; jiyeong1211@korea.kr; 4 Microbial Institute for Fermentation Industry, Sunchang 56048, Republic of Korea; tndusalal@naver.com; 5 Department of Food Science and Technology, Institute of Agriculture and Life Science, Gyeongsang National University, Jinju 52828, Republic of Korea; 6 National Institute of Crop Science, Rural Development Administration, Miryang 50424, Republic of Korea; emhee@korea.kr

**Keywords:** perilla seed powder, relative humidity, metabolite, machine learning

## Abstract

This study investigated the effects of relative humidity (RH) and storage period on the quality and metabolite profiles of perilla seed powder (PSP). PSP was stored for 12 weeks at RH levels ranging from 11% to 93%, and quality changes were monitored by assessing microbial growth, lipid oxidation, color, and metabolite profiles. Visual deterioration occurred rapidly above RH 69% due to microbial proliferation, becoming apparent after four weeks at RH 69% and after one week above RH 81%. In contrast, lipid oxidation, measured by acid and peroxide values, was significantly delayed at RH levels below 43%, whereas at 53% RH, the acid value increased 14.8-fold after 12 weeks compared to the initial level. Multivariate statistical analysis showed distinct metabolite patterns dependent on RH and storage period. Unsaturated fatty acids, such as linoleic and linolenic acids, decreased, whereas lysophosphatidylethanolamines (LPEs) and their oxidized derivatives, including hydroxylinolenic acid, increased by up to 167-fold at RH 53% after 12 weeks. Rosmarinic acid declined, whereas glycosylated phenolics, including rosmarinyl glucoside, increased. Multi-output regression models based on metabolite and quality traits effectively predicted RH and storage duration (R^2^ > 0.87, RMSE < 5.37), demonstrating their potential utility in monitoring storage conditions. These findings suggest that PSP should be stored under RH below 43% for no longer than four weeks to minimize quality degradation. This study provides new insights into RH-dependent metabolic responses in seed-based powders and offers a scientific basis for RH-controlled storage strategies to maintain product stability.

## 1. Introduction

Perilla (*Perilla frutescens*), commonly known as wild sesame, is extensively grown in East Asian countries, including Korea, China, Japan, Vietnam, and Thailand [1]. Its seeds are widely used as a food source, particularly for producing edible oil and seasoning, due to their distinctive flavor [2,3]. In addition, perilla seeds are rich in phenolic compounds, unsaturated fatty acids, and essential amino acids, providing various pharmacological benefits such as antitumor, anti-inflammatory, hypotensive, anti-atherosclerotic, and antidepressant activity, as well as anaphylactic shock mitigation [4,5,6,7]. However, improper storage conditions can significantly deteriorate the quality of perilla seeds due to the high content of easily oxidizable components such as polyunsaturated fatty acids (PUFAs), phenolic compounds, and tocopherols, which are highly susceptible to oxidative degradation [8].

Storage conditions, including relative humidity (RH), temperature, storage period, and exposure to light, play a crucial role in preserving seed quality by influencing phytochemical stability and inducing external stress-related changes [3,9]. Among these factors, RH is particularly critical in maintaining seed quality during storage, as inappropriate humidity levels can lead to adverse effects such as browning, oxidation, lipid peroxidation, and microbial proliferation [10]. Previous studies have explored the quality characteristics of whole perilla seeds during storage, assessing factors such as oil yield, physicochemical properties, oxidative stability, and microbial safety under a broad RH range of 11–93% [11]. Perilla seeds are frequently processed into powdered forms for use as seasonings [3]. Such processing markedly increases susceptibility to oxidative degradation, not only due to the increased surface area but also because milling disrupts cellular structures, exposing lipids to oxygen and accelerating oxidative reactions [12]. Notably, lipid oxidation during storage significantly alters the phytochemical composition, leading to a reduction in nutritional value, degradation of bioactive compounds, and the development of undesirable flavors and off-odors [13]. Despite these concerns, the effect of relative humidity on the quality and metabolic stability of PSP remains largely unexplored.

Recently, metabolomics, which utilizes highly sensitive analytical techniques such as nuclear magnetic resonance (NMR) and mass spectrometry (MS), has emerged as a powerful tool for investigating storage-induced alterations in nutrients and phytochemical profiles of seed-based food products such as hemp seeds, buckwheat seeds, sesame seeds, and chia seeds [14,15,16]. Previously, the quality changes in perilla seed powder (PSP) during storage were investigated, with oxidative stability, antioxidant activity, and chemical composition assessed at 25, 35, and 45 °C [3]. However, despite this characterization of the temperature effect, the RH effect on the phytochemical profiles of PSP remains largely unexplored.

Therefore, this study aims to investigate the impact of an RH of 11–93% on the quality of PSP by systematically evaluating parameters including microbial growth, lipid oxidation, color stability, and metabolite profile changes over a 12-week storage period.

## 2. Results and Discussion

### 2.1. Appearance Changes and Microbial Populations During Storage

**Figure 1:**
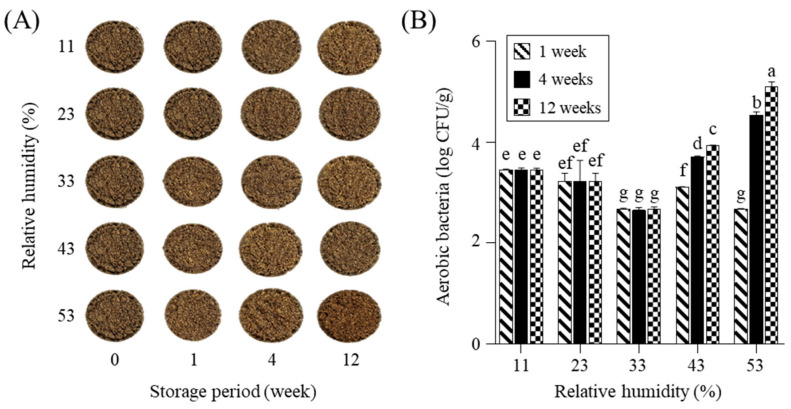
The appearances (**A**) and aerobic bacteria population (**B**) of perilla powder during storage for 12 weeks at different relative humidity. The different letters on each bar mean significantly different by Duncan’s test at *p* < 0.05.

The appearance of PSP exhibited minimal changes at RHs below 53% during the 12-week storage period (Figure 1A). In contrast, distinct changes occurred at RHs above 69% as early as week 1, attributable to the proliferation of fungi and yeast (Figure S1). Therefore, the storage experiment of PSP was carried out within an RH range of 11% to 53% for 12 weeks. Previous studies have demonstrated that excessive humidity accelerates microbial growth and quality deterioration in seed-based products [17].

Total aerobic bacterial counts in PSP showed no significant differences across RH levels from 11% to 33% over 12 weeks (Figure 1B). In contrast, an increase in bacteria was observed at RH levels above 43%. The increase in RH led to increased moisture content of samples, which favors the growth of microorganisms during storage [17,18]. Notably, yeast and mold were not detected, underscoring the diminished resistance of PSP to bacterial contamination under conditions of elevated RH compared to whole perilla seeds, as reported in our previous study [11]. This could be due to the dehulling process, which exposes the seed endosperm, thereby increasing its vulnerability to microbial contamination [19].

**Figure 2:**
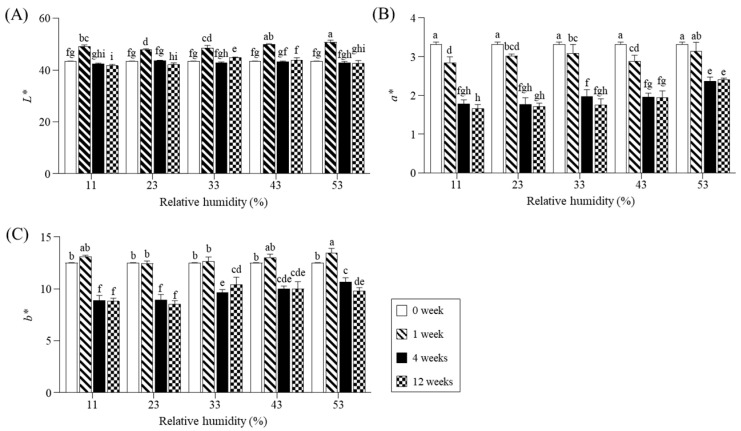
Color values of perilla powder during storage for 12 weeks at different relative humidity. (**A**), lightness (L*); (**B**), redness (a*); (**C**), yellowness (b*). The different letters on each bar at the same relative humidity denote a significant difference by Duncan’s test at *p* < 0.05. L*, lightness; a*, redness; b*, yellowness.

Regarding color changes (Figure 2), the L* values increased during the first week and decreased thereafter, with minimal influence from RH (Figure 2A). Unlike the L* values, the a* and b* values significantly decreased at all RH levels after 4 weeks of storage. Additionally, at weeks 4 and 12, the a* and b* values increased with increasing RH. RH may influence food color stability during storage by promoting oxidation reactions and enzyme activity [20]. However, lower RH levels had no significant effect on color changes. Our previous study also reported that the L*, a*, and b* values of whole perilla seeds remained stable at RHs of 11–53%, but changed significantly above 69% [11].

### 2.2. Lipid Oxidation in Perilla Seed Powders During Storage

**Figure 3:**
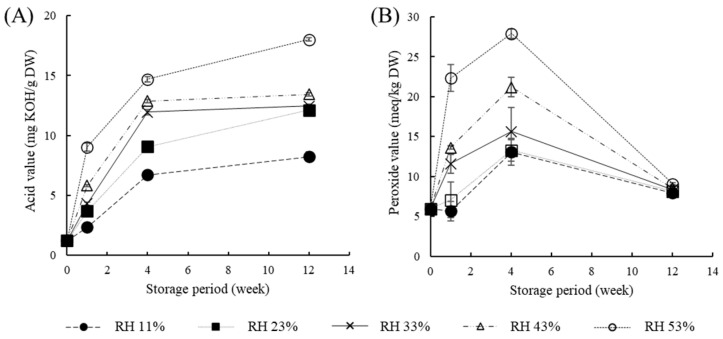
Acid (**A**) and peroxide values (**B**) of perilla powder during storage for 12 weeks at different relative humidity (RH).

Lipid oxidation in PSP during 12 weeks of storage was evaluated by AV and POV (Figure 3). The AV of PSP was 1.22 mg KOH/g DW at week 0 and increased sharply with increasing RH and longer storage periods (Figure 3A). At an RH of 53% after 12 weeks, AV was 14.8 times higher than that at week 0. The observed decrease in POV after 4 weeks could be attributed to the secondary oxidation process, in which peroxides formed initially are further degraded into secondary oxidation products such as aldehydes and ketones [21]. Although lipid oxidation is generally promoted at both very low (<30% RH) and high RH, the present study showed that POV values remained low at 11 and 23% and increased from 33 to 53. Nevertheless, some low-moisture matrices, such as kori-tofu [22] and corn oil [23], exhibit stable or prolonged lag phases under very low RH levels corresponding to water activity 0.05–0.20. Lipid oxidation may be influenced by processing conditions and sample matrix [24,25]. These results indicate that RH significantly affects lipid oxidation in PSP during storage. In general, higher RH increases moisture content, enhancing hydrolytic enzyme activity, respiration, and catalyst mobility, which leads to lipid oxidation via accumulation of free fatty acids and peroxides [26,27]. Additionally, previous studies reported that increased RH significantly enhances lipid oxidation in lipid-rich seeds such as walnut [28] and peanuts [29].

### 2.3. Metabolomic Analysis

**Figure 4:**
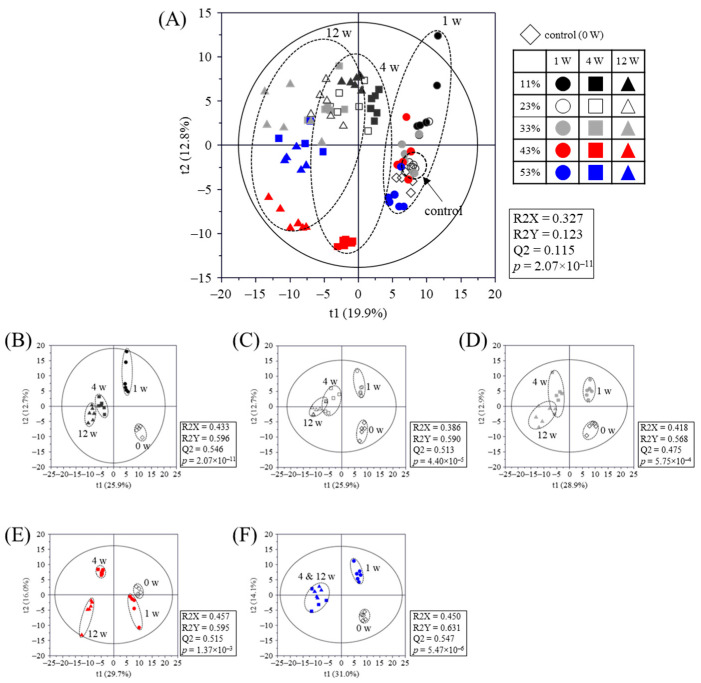
Partial least-squares discriminant analysis (PLS-DA) score plots of perilla seed powder metabolites analyzed by UPLC-Q-TOF MS and GC-MS and their quality parameters. The goodness of fit of the PLS-DA model was evaluated by R2X, R2Y, Q2, and *p*-value and validated using cross-validation with a permutation test. (**A**) PLS-DA score plot of perilla powder metabolites stored at different relative humidity (RH) for 1 to 12 weeks; (**B**) RH at 11%; (**C**) 23%; (**D**) 33%; (**E**) 43%; (**F**) 53%.

The metabolite profiles in PSP samples stored at different RHs were analyzed by GC-MS and UPLC-Q-TOF MS (Figure S2), and discrimination of PSP samples was visualized using PLS-DA score plots (Figure 4). Statistical parameters, including goodness of fit (R2X = 0.327–0.457; R2Y = 0.123–0.631), predictability (Q2 = 0.115–0.547), *p*-value (1.37 × 10^−3^–2.07 × 10^−11^), and cross validation via permutation test (*n* = 200) (Figure S3), indicated that the PLS-DA models were statistically acceptable.

In the PLS-DA score plot for all samples (Figure 4A), PSP samples stored at 43% RH for 4 and 12 weeks showed distinct clusters, indicating notable metabolomic differences compared to samples stored under other conditions. In addition, PLS-DA score plots at each RH level were clearly separated by the storage periods along t(1) and t(2) (Figure 4B–F).

**Figure 5:**
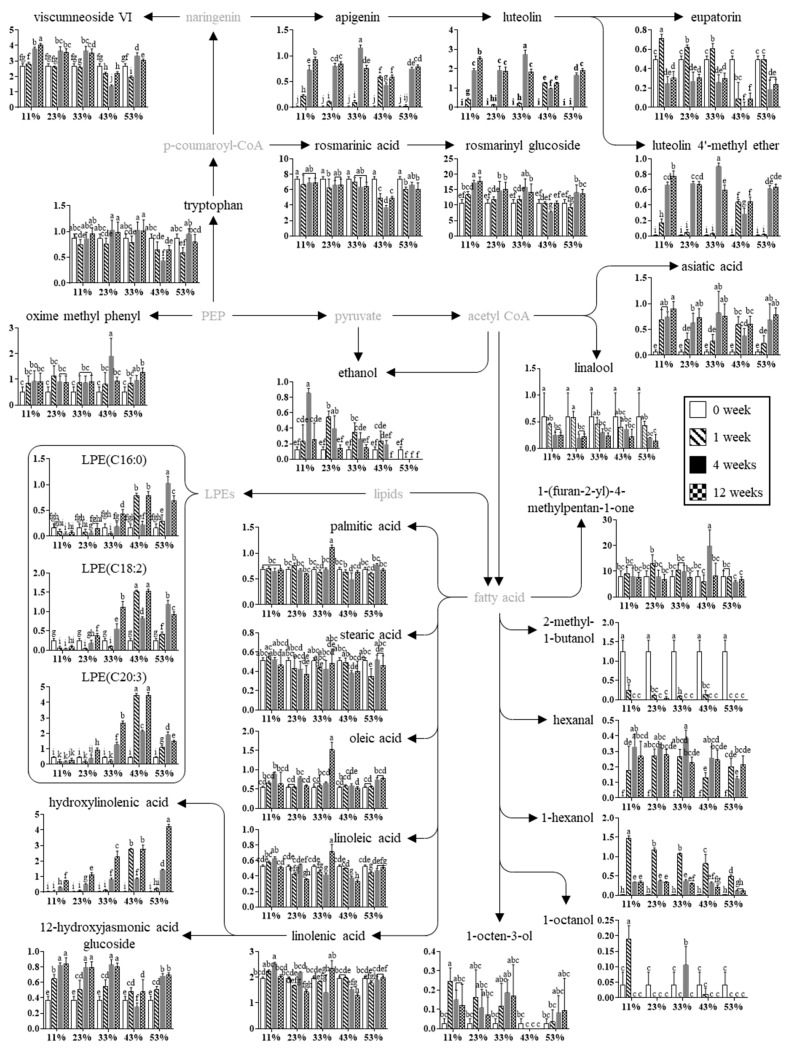
Metabolomic pathway of perilla metabolites associated with storage at different relative humidity. Metabolites were analyzed by UPLC-Q-TOF MS and GC/MS. The *Y*-axis means the normalized chromatogram intensity, and the *X*-axis means the relative humidity (%). The different letters on each bar denote a significant difference by Duncan’s test at *p* < 0.05. LPE, lysophosphatidylethanolamine; PEP, phosphoenolpyruvate.

The major metabolites involved in the discrimination between sample groups in PLS-DA score plots were identified based on variable importance in the projection (VIP) and *p*-values (Tables S1 and S2). Twenty-eight metabolites were identified, including nine lipids (palmitic acid, stearic acid, oleic acid, linoleic acid, linolenic acid, hydroxylinolenic acid, and three lysophosphatidylethanolamines [LPEs]), seven phenolic compounds (rosmarinyl glucoside, viscumneoside VI, rosmarinic acid, eupatorine, luteolin, apigenin, and luteolin 4′-methyl ether), nine volatile compounds [ethanol, hexanal, 2-methyl-1-butanol, 1-hexanol, 1-octen-3-ol, linalool, 1-octanol, oxime methoxy phenyl, and 1-(furan-2-yl)-4-methyl pentan-1-one], 1 amino acid (tryptophan), 1 triterpenoid (asiatic acid), and 1 fatty acyl glucoside (12-hydroxyjasmonic acid glucoside). These metabolites were selected using VIP ≥ 0.84 and *p* < 0.05. Notably, similar metabolite profiles were identified in our previous study on PSP storage at different temperatures (25, 35, and 45 °C) for 8 weeks [3]. These metabolites reflected distinct oxidative and metabolic responses to RH variation [25]. In particular, storage under higher RH conditions was associated with elevated levels of oxidized lipids and volatiles, indicating accelerated lipid peroxidation and degradation of unsaturated fatty acids [5,24]. This trend was further examined through pathway analysis and comparison of the relative abundances of identified metabolites (Figure 5).

### 2.4. Metabolomic Pathway and Relative Abundances of Identified Metabolites

The metabolomic pathway of PSP during storage at different RHs was proposed, and the relative abundances of the identified metabolites were compared (Figure 5). The lipid oxidation and changes in secondary metabolites were the major metabolic processes in PSP during storage.

During storage, the lipid profiles of seeds and nuts, which provide energy for the developing seedling during germination, change significantly. Changes in lipid metabolism during storage are regulated by various enzymes and transcription factors that control the biosynthesis, degradation, and remodeling [30]. Lipid metabolism is also influenced by environmental factors such as temperature, humidity, and light, which affect the stability and composition of stored lipids [31]. In this study, palmitic acid remained largely unchanged across RH conditions during 12 weeks of storage, except for a notable increase at 33% RH after 4 weeks. Stearic acid showed a slight decline at low RH levels (11% and 23%), but overall changes were minimal. The relative stability of saturated fatty acids compared to polyunsaturated fatty acids suggests their lower susceptibility to oxidative degradation, consistent with previous findings regarding their lower reactivity. In contrast, the levels of unsaturated fatty acids, including oleic, linoleic, and linolenic acids, exhibited a U-shaped pattern across RH conditions, being relatively high at both low (11–23%) and high (43–53%) humidity, but lower at moderate RH (33%). Notably, the 33% RH sample stored for 4 weeks deviated from this pattern, showing the highest levels among all conditions. This increase can be linked to decreased oxidative degradation and better preservation of free fatty acids in low-moisture environments. A previous study reported that low RH (particularly 33%) enhances the oxidative stability of perilla seeds, corroborating the hypothesis that dry conditions inhibit lipid peroxidation and promote the retention of unsaturated fatty acids [10,11]. Additionally, LPEs (C16:0, C18:2, and C20:3) and hydroxylinolenic acid increased markedly with increasing RH and storage time. The substantial accumulation of hydroxylinolenic acid, especially between 33% and 53% RH, where its levels after 12 weeks were up to 167-fold higher than initial levels, indicates that oxidation processes are strongly enhanced under humid conditions and longer storage time. This extreme increase suggests advanced lipid peroxidation, which may compromise both the sensory attributes and nutritional value of the product [24,32]. Polyunsaturated fatty acids such as linoleic and linolenic acids are more susceptible to oxidative degradation with increasing RH and storage duration [32]. This condition may also enhance the oxidation of LPEs and linolenic acid, resulting in increased levels of secondary products such as hydroxylinolenic acid [33]. Together, these findings suggest that while moderate RH (around 33%) may transiently stabilize unsaturated fatty acids, higher RH levels promote their degradation through both hydrolytic and oxidative pathways. Moreover, 12-hydroxyjasmonic acid glucoside, a jasmonate-related metabolite derived from linolenic acid, exhibited a clear time-dependent accumulation pattern across all RH conditions, although some intermediate suppression was observed at 43% RH. These results suggest that the formation of this compound is primarily driven by storage duration, likely through sustained oxidative or enzymatic conversion of linolenic acid under long-term storage conditions [34].

In addition to lipids, the levels of volatile compounds derived from lipid oxidation, including 2-methyl-1-butanol, hexanal, 1-hexanol, 1-octen-3-ol, and ethanol, were markedly influenced by storage time and RH conditions. 2-Methyl-1-butanol, which was initially present at week 0, declined sharply by week 1 and was not detected from week 4 onward across all RH conditions. In contrast, hexanal, 1-hexanol, and 1-octen-3-ol, which were either absent or present at trace levels initially, increased with prolonged storage, though their accumulation was generally suppressed under higher RHs. 1-Hexanol reached its maximum level at week 1, then declined rapidly, with minimal changes observed between weeks 4 and 12. The level of 1-octen-3-ol was nearly undetectable at 43% RH but showed a resurgence at 53% RH. Ethanol levels increased until weeks 1 or 4 but dropped substantially by week 12. A clear decreasing trend was also observed with increasing RH, and ethanol was undetectable in samples stored for 12 weeks at 43% RH and in all samples stored at 53% RH from week 1 onward. These changes in volatile and lipid-related metabolites can be largely attributed to lipid autooxidation, which is likely accelerated in powdered perilla seed due to its high surface area and abundance of polyunsaturated fatty acids such as linolenic, linoleic, and oleic acids [24,35]. Under storage conditions with RH ≤ 53%, microbial activity is expected to be minimal, suggesting that these metabolic alterations primarily result from enzymatic and oxidative processes rather than microbial degradation. Volatile compounds such as hexanal, 1-hexanol, and 1-octen-3-ol, which are typical breakdown products of hydroperoxides formed during the oxidation of unsaturated fatty acids, showed significant accumulation during storage, especially under low RH conditions. At higher RH, however, the levels of these volatiles did not increase markedly, likely due to their further degradation into more advanced oxidation products or their loss through volatilization [24]. This pattern aligns with the observed decline in POV after 4 weeks, suggesting that hydroperoxides formed at early stages were decomposed into volatiles and other secondary products as storage progressed. The generation of hexanal has been previously associated with the oxidation of linoleic acid in systems such as rapeseed oil [21] and pine needle oil [36], while 1-octen-3-ol is a known product of linoleic acid oxidation and contributes to mushroom- or beany-like aromas [37]. The varying patterns of 1-octen-3-ol across RH levels suggest that oxidative pathways are differentially regulated depending on moisture availability. Additionally, ethanol levels increased during early storage but declined at week 12, particularly under high RH conditions, potentially due to volatilization or consumption during oxidation [38]. The concurrent rise in hydroxylinolenic acid and lysophosphatidylcholines (LPCs; C16:0, C18:2, C20:3) further supports the involvement of phospholipid oxidation. LPCs are known to arise from the hydrolytic cleavage and oxidative degradation of membrane phospholipids, indicating membrane destabilization and lipid peroxidation during storage [21]. Together, these findings suggest that the formation and degradation of lipid-derived volatiles and oxidized lipid intermediates in PSP are primarily governed by storage duration and oxidative reactions, with RH modulating the rate and extent of these changes.

The profiles of secondary metabolites in perilla seed were significantly affected by storage time and RH conditions, with 43% RH consistently associated with the greatest reductions in metabolite levels. The levels of major metabolites such as viscumneoside VI, rosmarinyl glucoside, and eupatorin, which were detected at the initial stage, generally increased with prolonged storage. In contrast, rosmarinic acid and linalool exhibited a slight decreasing trend throughout the 12-week storage period. Minor or initially undetected compounds, including apigenin, luteolin, luteolin 4′-methyl ether, and asiatic acid, showed marked increases during storage, regardless of RH conditions, except for a noticeable reduction at 43% RH. The observed changes in secondary metabolites are likely attributed to enzymatic activity during storage, as the perilla seed was stored in powdered form at RH levels up to 53%, which are generally unsuitable for microbial growth [3]. Compounds such as apigenin, luteolin, and asiatic acid, initially undetected or minimal, increased markedly, suggesting enzymatic conversion from precursor metabolites. In contrast, the decline in rosmarinic acid and linalool may reflect their susceptibility to degradation. Notably, reductions were most evident at 43% RH, possibly due to stress-induced enzymatic reactions at this intermediate moisture level [39]. These findings are meaningful because many of the altered compounds have recognized physiological and industrial value. Flavonoids such as apigenin and luteolin possess a broad spectrum of biological activities, including antioxidant, anti-inflammatory, anticancer, and neuroprotective effects [40,41]. Asiatic acid exhibits hepatoprotective and anti-inflammatory properties by modulating lipid metabolism and cellular stress responses [2], while linalool contributes to the aroma of the product and is widely used in the fragrance and cosmetics industries [42]. Thus, controlling storage conditions may enhance or preserve the functional and industrial potential of perilla seed metabolites.

### 2.5. Prediction of Storage RH and Weeks Using Regression Models

**Figure 6:**
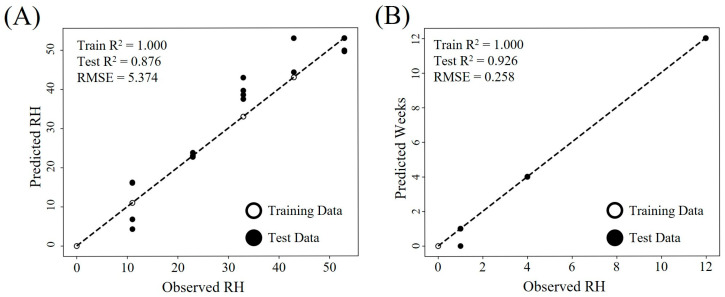
Scatter plots of predicted and observed storage relative humidity (RH, (**A**)) and duration (weeks, (**B**)) for the eXtreme Gradient Boosting (XGBoost) model. The model was trained on scaled metabolite profiles and quality parameters of perilla seed powder. Open and filled circles represent training and test data, respectively. Model performance was evaluated using the coefficient of determination (R^2^) and root mean square error (RMSE), where higher R^2^ and lower RMSE values indicate better predictive accuracy.

Machine learning-based regression models were employed to predict storage RH and duration using the combined dataset of metabolite profiles and quality indicators, such as color values, aerobic bacterial counts, and lipid oxidation indices. Four multi-output regression models such as Random Forest, Support Vector Machine (SVM), eXtreme Gradient Boosting (XGBoost), and k-nearest neighbors (KNN) were evaluated for their predictive performance (Figure 6 and Figure S4). Random Forest and XGBoost showed the highest accuracy for the prediction of storage RH and period with coefficient of determination (R^2^) values of 0.885 and 0.876 for RH prediction and 0.756 and 0.996 for storage period prediction, respectively. In particular, XGBoost demonstrated exceptional performance for storage period prediction, yielding a root mean squared error (RMSE) of 0.258 and exhibiting strong predictive power and robustness in modeling complex patterns from metabolites and quality datasets. On the other hand, SVM and KNN models exhibited relatively low prediction accuracy, especially under intermediate RH levels or longer storage periods, likely due to limitations in modeling nonlinear and heterogeneous distributions [43]. Variables with feature importance values greater than 0.15 were deemed significant, with LPE (C20:3) (0.617) and 12-hydroxyjasmonic acid glucoside (0.150) recognized as primary predictors for RH, alongside hydroxylinolenic acid (0.758) and POV (0.198) for storage duration. These results underscore the essential functions of lipid oxidation products and jasmonate-related metabolites in evaluating storage conditions [32,34].

Predicting the storage duration and relative humidity of PSP using metabolomics may offer an effective approach to quality assessment, particularly for powdered products like PSP that exhibit limited visual differences during storage (Figure 1). The integration of metabolomics with machine learning has been applied across various domains, including quality control, nutritional studies, and food adulteration detection [44]. In the context of food storage, this combined approach has also been effectively used to predict quality deterioration in visually subtle crops such as sweetcorn seed [45] and strawberries [46], enabling early detection of internal quality changes. Additionally, further optimization and training of these machine learning models could enhance predictive accuracy, offering a promising strategy for the comprehensive quality management of powdered seed products [45].

## 3. Materials and Methods

### 3.1. Sample Preparation

Perilla seeds (cultivar Dayu), harvested in 2020 using a mechanical combine harvester, were obtained from the Chungbuk Agricultural Research and Extension Services of Korea. Perilla seeds were vacuum-sealed in sterile polyethylene pouches, then ground using a vacuum-nitrogen displacement grinding device [47] (−80 mesh) and passed through an 80-mesh sieve. The PSP samples were then stored at different RH levels using an airtight container at 25 °C. The desired RH conditions (11, 23, 33, 43, 53, 69, 81, and 93%) were achieved using saturated solutions of LiCl, KCH_3_CO_2_, MgCl_2_, K_2_CO_3_, Mg(NO_3_)_2_, KI, (NH_4_)_2_SO_4_, and KNO_3_ (Sigma-Aldrich, St. Louis, MO, USA), respectively, prepared at 25 °C and placed on sterile aluminum dishes within the containers. The PSP samples were stored for 12 weeks under each condition, and some fresh portions were immediately used to analyze the microbial population, color, and volatile compounds. Other portions were freeze-dried and stored at −18 °C until use.

### 3.2. Color, Microbial Population, and Lipid Oxidation

Color values (L*, a*, and b*) of the PSP samples were measured using a CR-301 Chroma Meter (Konica Minolta, Osaka, Japan) by positioning the sensor head directly onto the sample surface, with data recorded in triplicate from different locations. The microbial population of the samples was measured as follows: 5 g of each PSP sample was combined and mixed with 45 mL of 0.85% (*w/v*) sodium chloride solution in a sterile filter bag and homogenized by a stomacher (Seaward, Peterlee, UK) for 2 min. Serial dilutions were executed using the same solution, followed by plating 1 mL aliquots onto 3M Petrifilm™ Aerobic Count Plates and Yeast and Mold Count Plates (3M Company, St. Paul, MN, USA). The plates were incubated at 35 °C for 48 h for aerobic bacteria, and at 25 °C for 120 h for yeast and mold. After incubation, colonies on the Petrifilm were counted and expressed as log CFU/g. The AV and POV of samples were determined according to the Association of Official Analytical Chemists [48]. AV and POV results were expressed as mg KOH/g dry weight (DW) and meq/kg DW, respectively.

### 3.3. GC-MS Analysis

GC-MS-based metabolomic analysis of the samples was performed according to a previously described method with minor modifications [3]. The lyophilized sample was homogenized with 0.5 M KOH/MeOH at 90 °C for 15 min. Boron trifluoride–methanol (BF_3_-MeOH) was then added to the extract and reacted at 90 °C for 2 min. Hexane was added to extract free fatty acids. After centrifugation, the hexane layer was derivatized at 70 °C for 20 min using N, O-bis (trimethylsilyl)trifluoroacetamide (BSTFA) containing 5-cholestane as an internal standard (IS). The derivatized samples were injected into a GC-2010 plus apparatus (Shimadzu, Tokyo, Japan) equipped with a DB-5MS capillary column (30 m × 0.25 mm i.d., 0.25 μm film thickness, Agilent J&W, Santa Clara, CA, USA) with a split ratio of 1:10. The oven temperature was held at 80 °C for 2 min, then ramped to 320 °C at 10 °C/min, and held at 320 °C for 3 min. Volatile compounds of perilla powder were analyzed using a solid-phase microextraction (SPME)-GC-MS. A fresh sample and 1 mL of distilled water containing 2-methyl-1-pentanol as an IS were placed in a septum cap vial and heated at 65 °C for 10 min. Subsequently, headspace volatile compounds were absorbed with an SPME fiber (50/30 μm DVB/CAR/PDMS Stableflex, Sigma-Aldrich) at 65 °C for 10 min. The fiber was then desorbed into a GC equipped with a DB-WAX capillary column (30 m × 0.25 mm i.d., 0.25 um film thickness, Agilent J&W). The oven program was set as follows: 40 °C for 3 min, ramp to 90 °C at 5 °C/min, then ramp to 230 °C at 19 °C/min and hold for 5 min. Helium was used as the carrier gas at 1 mL/min. Injector, ion source, and interface temperatures were 200, 230, and 280 °C, respectively. Eluted metabolites were detected using a GC/MS-TQ 8030 device (Shimadzu) with electron ionization (EI) mode at 70 eV. MS spectra were acquired in full-scan mode from *m/z* 45 to 550.

### 3.4. UPLC-Q-TOF-MS Analysis

UPLC-Q-TOF-MS-based metabolomic analysis of the samples was performed according to a previously described method with minor modifications [3]. The lyophilized samples were homogenized using 80% (*v/v*) methanol containing abscisic acid as an IS. After centrifugation, the supernatants were injected into an Acquity UPLC BEH C18 column (2.1 mm × 100 mm, 1.7 μm, Waters, Milford, MA, USA) equilibrated with distilled water containing 0.1% formic acid. Metabolites were eluted with a linear gradient of acetonitrile containing 0.1% formic acid at 0.35 mL/min. Eluted metabolites were analyzed using a Q-TOF-MS system (Xevo, G2-S-QTOF; Waters) with negative electrospray ionization mode. The optimal MS conditions were set at a sampling cone voltage of 40 V, a capillary voltage of 3 kV, a desolvation temperature of 200 °C, a source temperature of 100 °C, and a desolvation gas flow rate of 700 L/h. MS data were collected in full-scan mode of *m/z* 50–1500 at a scan time of 0.2 s. Leucine-enkephalin ([M − H] = 554.2615) was used as the lock mass reference. The quality control (QC) samples prepared by mixing all samples were analyzed after every ten injections. MS/MS spectra were obtained using a collision energy ramp of 10–40 eV.

### 3.5. Data Processing

The GC-MS data were aligned and normalized to the IS. Metabolites analyzed by GC-MS were identified by retention indices (RIs) calculated using C8-C40 n-alkanes and GC-MS databases (NIST 11 and Wiley 9 mass spectral libraries). The UPLC-Q-TOF MS data were processed using MarkerLynx software ver. 4.2. (Waters) and normalized to the IS. Metabolites were identified using Chemspider online databases via UNIFI software ver. 3.13.0. (Waters).

### 3.6. Prediction Models for Storage Relative Humidity and Duration

Python (ver. 3.12.7) with scikit-learn (ver. 1.5.1) and XGBoost (eXtreme Gradient Boosting, ver. 3.0.2) libraries was used to develop predictive models for estimating the storage RH and period (weeks) of PSP based on metabolite profiles and quality characteristics. Prior to modeling, missing values were imputed using the mean of each variable, and extreme outliers were removed based on principal component analysis (PCA). All features were then scaled using the StandardScaler function. The dataset was randomly divided into training (70%) and test (30%) sets with stratification by RH classes. Four multi-output regression models, Random Forest, SVM, XGBoost, and KNN, were used to simultaneously predict RH and weeks. Model hyperparameters were manually tuned: 200 trees and a max depth of 10 for Random Forest, RBF kernel for SVM, learning rate of 0.1 and a max depth of 5 for XGBoost, and 5 neighbors for KNN [44]. Model performance was evaluated using the R^2^ and RMSE for each output, and observed versus predicted scatter plots were generated to visualize model accuracy [47].

## 4. Conclusions

This study evaluated the effects of storage period and RH on the quality of PSP by monitoring changes in microbial growth, lipid oxidation, color stability, and metabolite profiles over 12 weeks at RH levels ranging from 11% to 93%. Visual quality rapidly declined above 69% RH due to microbial growth, while lipid oxidation remained low for up to 4 weeks at RH below 43%. Metabolite profiling revealed RH- and time-dependent changes, including decreases in unsaturated fatty acids and increases in lipid oxidation markers such as hydroxylinolenic acid and LPEs. Flavonoids and triterpenoids also showed notable RH sensitivity, highlighting their potential as quality indicators. A machine learning-based regression model using these variables demonstrated high predictive performance for both RH (R^2^ = 0.885 and RMSE = 5.176) and storage period (R^2^ = 0.926 and RMSE = 0.258). Although this study was limited to a single cultivar and harvest batch, it underscores the importance of humidity control in PSP, identifying RH < 43% and storage duration <4 weeks as critical conditions for quality preservation. These findings also provide a scientific basis for RH-controlled storage strategies and quality prediction in seed-derived powders.

## Data Availability

The original contributions presented in this study are included in the article/Supplementary Materials. Further inquiries can be directed to the corresponding author.

## Supplementary Materials

The following supporting information can be downloaded at https://www.mdpi.com/article/10.3390/molecules30183662/s1: Figure S1: Appearance of perilla powder after 12 weeks of storage under high relative humidity conditions (>69%); Figure S2: Representative chromatograms of perilla powder analyzed by UPLC-Q-TOF MS (A) and GC/MS (B). Global metabolites were analyzed by UPLC-Q-TOF MS, while fatty acids and phytosterols were analyzed by GC-MS; Figure S3: Cross-validation of PLS-DA models (Figure 4) for PSP samples stored at different relative humidities (RHs) for 12 weeks. PLS-DA score plots were cross-validated with a permutation test (*n* = 200); Figure S4: Scatter plots of predicted and observed storage relative humidity (RH, A) and duration (weeks, B) for different prediction models. The models were trained on scaled metabolite profiles and quality parameters of perilla seed powder; Table S1: Identification of major perilla metabolites analyzed by UPLC-Q-TOF MS; Table S2: Identification of major perilla metabolites analyzed by GC-MS.
