# Supplementary material for: Effect of Relative Humidity on Quality and Metabolite Profiles of Perilla frutescens Seed Powder During Storage
Source: Molecules. 2025 Sep 9;30(18):3662. doi: 10.3390/molecules30183662 (PMC12472856; doi:10.3390/molecules30183662)
Supplement: Supplementary file 1 [file molecules-30-03662-s001.zip › molecules-3761482-supplementary.pdf]

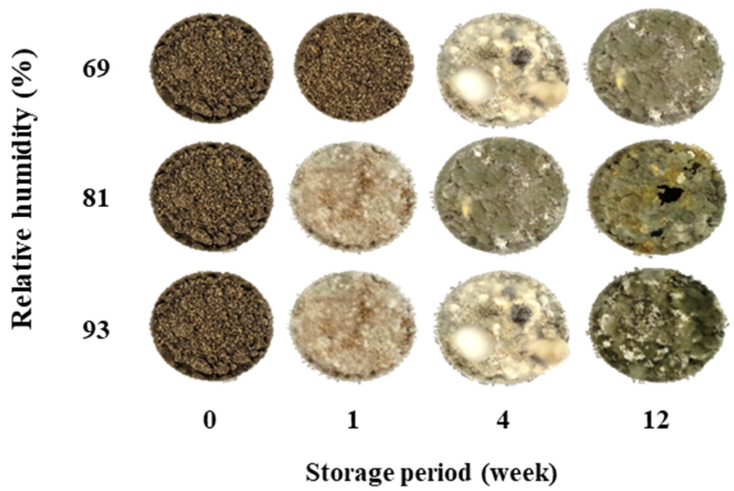

Figure S1. The appearances of perilla powder during storage for 12 weeks at high relative humidity (>69%).

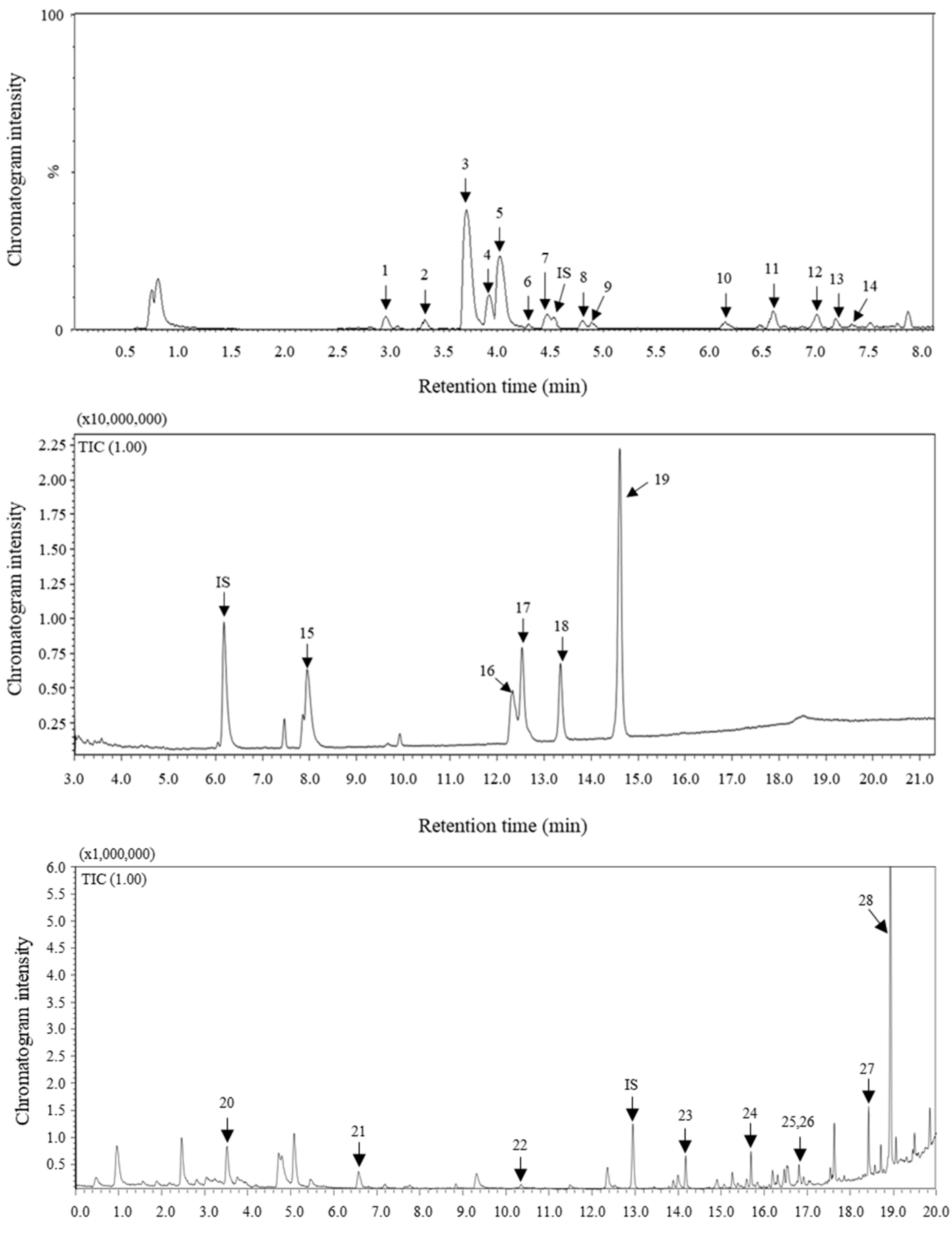

Figure S2. Representative chromatograms of perilla powder analyzed by UPLC-Q-TOF MS (A) and GC/MS (B). Global metabolites were analyzed by UPLC-Q-TOF MS, while fatty acids and phytosterols were analyzed by GC-MS. 1, tryptophan; 2, 12-hydroxyjasmonic acid glucoside; 3, rosmarinyl glucoside; 4, viscumneoside VI; 5, rosmarinic acid; 6, eupatorin; 7, luteolin; 8, apigenin; 9, luteolin 4'-methyl ether; 10, asiatic acid; 11, lysophosphatidylethanolamine (LPE) (C20:3); 12, LPE(C18:2); 13, hydroxylinolenic acid; 14, LPE(C16:0); 15, palmitic acid; 16, stearic acid; 17, oleic acid; 18, linoleic acid; 19, linolenic acid; 20, ethanol; 21, hexanal; 22, 2-methyl-1-butanol; 23, 1-hexanol; 24, 1-octen-3-ol; 25, linalool; 26, 1-octanal; 27, oxime methoxy phenyl; 28, 1-(furan-2-yl)-4-methylpentan-1-one.

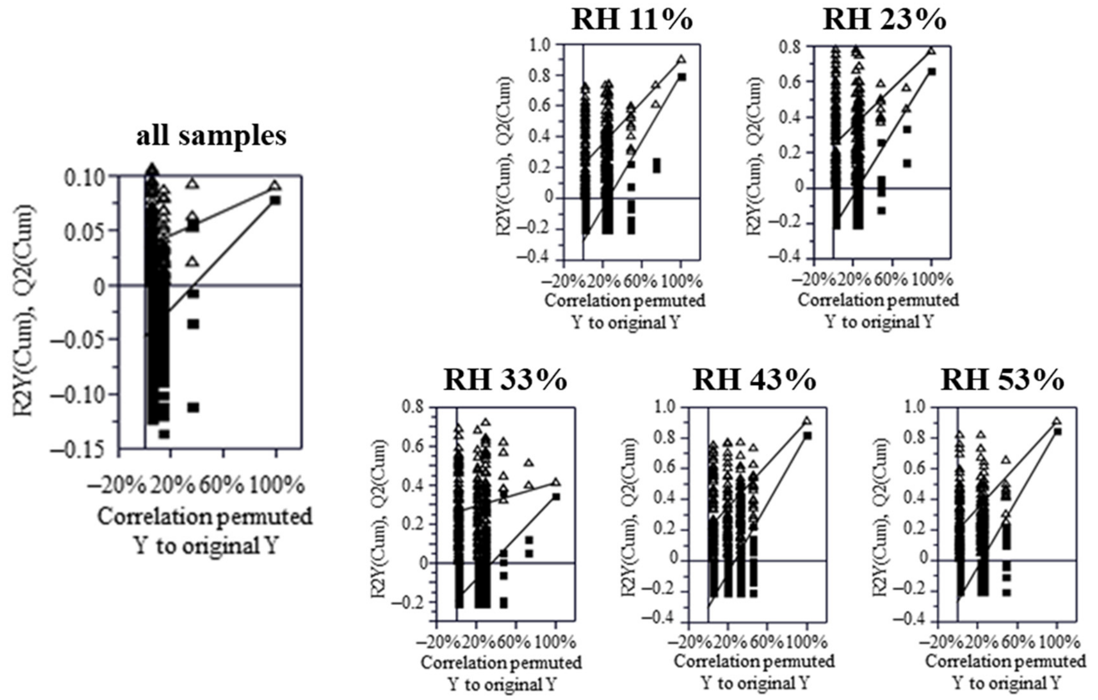

Figure S3. Cross-validation of PLS-DA models (**Fig. 4**) for PSP samples stored at different relative humidities (RHs) for 12 weeks. PLS-DA score plots were cross-validated with a permutation test (n = 200).

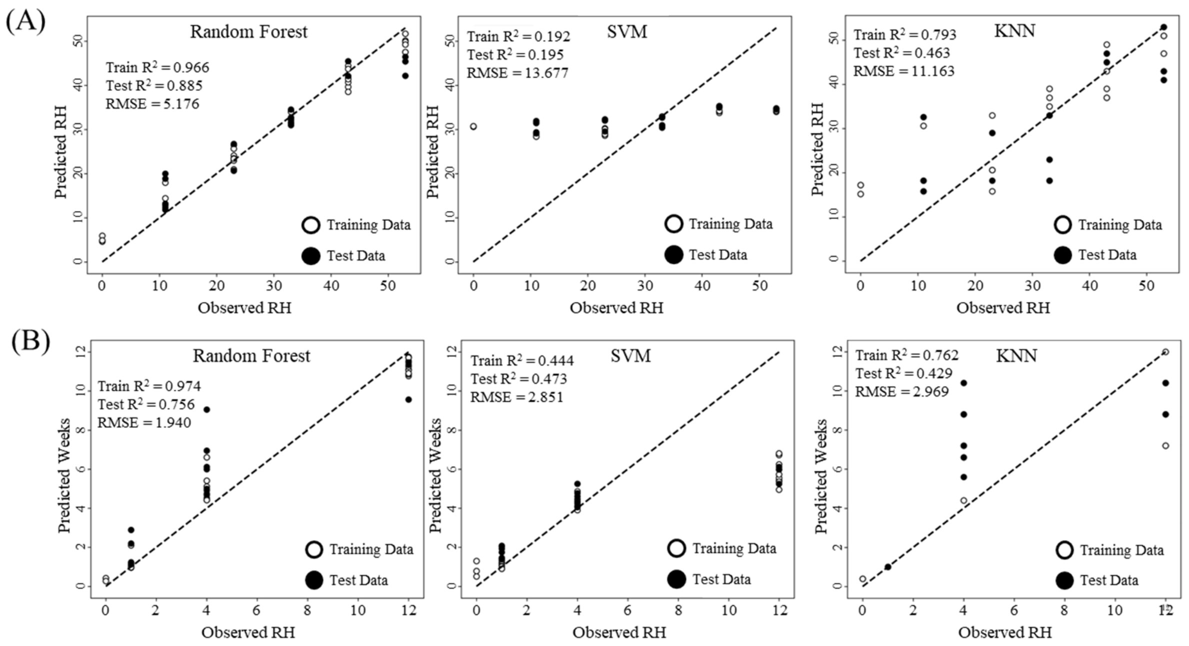

Figure S4. Scatter plots of predicted and observed storage relative humidity (RH, A) and duration (weeks, B) for different prediction models. The models were trained on scaled metabolite profiles and quality parameters of perilla seed powder. Open and filled circles represent training and test data, respectively. Model performance was evaluated using the coefficient of determination (R^2) and root mean square error (RMSE), where higher R^2 and lower RMSE values indicate better predictive accuracy. SVM, support vector machine; KNN, k-nearest neighbors; RMSE, root mean square error.

Table S1. Identification of major perilla metabolites analyzed by UPLC-Q-TOF MS.

RT	Metabolites	Exact mass (M-H)	MS error (mDa)	MS Fragments	*p*-value[a]	VIP[b]
2.94	tryptophan	203.0812	0.005	116	1.87×10^{-11}	1.49
3.30	12-hydroxyjasmonic acid glucoside	387.1648	0.013	281, 179, 116	4.30×10^{-26}	1.86
3.70	rosmarinyl glucoside	521.1311	-0.01	359, 323, 161	1.07×10^{-17}	1.73
3.90	viscumneoside VI	505.1375	-0.023	323, 161	9.97×10^{-46}	1.97
4.01	rosmarinic acid	359.0764	0.009	313, 179, 161, 133	3.60×10^{-11}	1.29
4.29	eupatorin	343.0806	0.017	493, 359, 161, 133	5.34×10^{-32}	1.37
4.45	luteolin	285.0395	0.026	116	1.04×10^{-63}	1.67
4.79	apigenin	269.0443	0	285, 116	4.87×10^{-64}	1.68
4.87	luteolin 4'-methyl ether	299.0548	0.013	284, 116	2.41×10^{-59}	1.70
6.13	asiatic acid	487.3429	0	533, 309, 116	2.55×10^{-12}	1.40
6.58	LPE(C20:3)	562.3144	-0.205	502, 474	1.38×10^{-67}	1.64
6.97	LPE(C18:2)	476.2772	0.011	564, 504	3.50×10^{-57}	1.66
7.18	hydroxylinolenic acid	293.2104	0.018	831, 515, 469, 275	8.63×10^{-64}	1.43
7.33	LPE(C16:0)	540.3292	0.004	831, 452, 293	6.81×10^{-39}	1.34

RT, retention time; VIP, variable importance in the protection; LPE, lysophosphatidulethanolamine.

[a]*p*-values were analyzed by ANOVA with Duncan's test.

Table S2. Identification of major perilla metabolites analyzed by GC-MS

	**RT**	**Metabolites**	***p*-value**	**VIP**
fatty acids	7.95	palmitic acid	4.68×10^{-31}	1.45
	12.31	stearic acid	0.002	0.84
	12.51	oleic acid	4.20×10^{-30}	1.31
	13.32	linoleic acid	2.94×10^{-40}	1.65
	14.59	linolenic acid	1.55×10^{-20}	1.47
volatile compounds	3.51	ethanol	4.00×10^{-10}	1.00
	6.57	hexanal	1.23×10^{-5}	0.75
	10.21	2-methyl-1-butanol	3.90×10^{-16}	0.76
	14.17	1-hexanol	1.48×10^{-19}	1.19
	15.62	1-octen-3-ol	0.024	1.05
	16.80	linalool	0.011	1.21
	16.92	1-octanal	1.85×10^{-11}	0.75
	18.42	methoxy-phenyl-oxime	0.013	0.78
	18.93	1-(furan-2-yl)-4-methyl pentan-1-one	5.23×10^{-4}	0.93

RT, retention time; RI, retention indices; VIP, variable importance in the protection.

p-values were analyzed by ANOVA with Duncan's test.
